# Determination of the volume-specific surface area by using transmission electron tomography for characterization and definition of nanomaterials

**DOI:** 10.1186/1477-3155-9-17

**Published:** 2011-05-11

**Authors:** Elke AF Van Doren, Pieter-Jan RH De Temmerman, Michel Abi Daoud Francisco, Jan Mast

**Affiliations:** 1 EM-unit, CODA-CERVA, Groeselenberg 99, Brussels, Belgium

## Abstract

**Background:**

Transmission electron microscopy (TEM) remains an important technique to investigate the size, shape and surface characteristics of particles at the nanometer scale. Resulting micrographs are two dimensional projections of objects and their interpretation can be difficult. Recently, electron tomography (ET) is increasingly used to reveal the morphology of nanomaterials (NM) in 3D. In this study, we examined the feasibility to visualize and measure silica and gold NM in suspension using conventional bright field electron tomography.

**Results:**

The general morphology of gold and silica NM was visualized in 3D by conventional TEM in bright field mode. In orthoslices of the examined NM the surface features of a NM could be seen and measured without interference of higher or lower lying structures inherent to conventional TEM. Segmentation by isosurface rendering allowed visualizing the 3D information of an electron tomographic reconstruction in greater detail than digital slicing. From the 3D reconstructions, the surface area and the volume of the examined NM could be estimated directly and the volume-specific surface area (VSSA) was calculated. The mean VSSA of all examined NM was significantly larger than the threshold of 60 m^2^/cm^3^.

The high correlation between the measured values of area and volume gold nanoparticles with a known spherical morphology and the areas and volumes calculated from the equivalent circle diameter (ECD) of projected nanoparticles (NP) indicates that the values measured from electron tomographic reconstructions are valid for these gold particles.

**Conclusion:**

The characterization and definition of the examined gold and silica NM can benefit from application of conventional bright field electron tomography: the NM can be visualized in 3D, while surface features and the VSSA can be measured.

## Background

The number based size distribution of a material and the features of its surface are predominant criteria to classify it as a NM [1,2]. TEM remains an important technique to measure the size and surface topography of materials at the nanometer level. Because the resulting micrographs are two-dimensional projections of the studied objects, their interpretation can be difficult, particularly when the particles are complex, agglomerated or lack symmetry. In such cases, fine ultrastructural details are blurred due to superposition of projected features. In addition, parameters like the surface area and volume of NM are not accessible by conventional TEM, while the approach to measure the thickness of NM along the projection direction by analyzing focal series in TEM assumes a relatively simple structure [3]. Recently, as data acquisition, alignment and reconstruction software evolves to be more user-friendly; ET is increasingly used to reveal the morphology and to evaluate the three-dimensional characteristics of NP and nanoparticle ensembles [4,5].

To include also aggregates and agglomerates of primary particles and complex multi-component particles with external dimensions larger than the arbitrarily specified upper size limit of 100 nm, the VSSA is proposed as a complementary qualifier to distinguish a nanostructured material from a non-nanostructured material [1]. The European Commission [2] proposes to define a material as a NM when it has a specific surface area by volume greater than 60 m^2^/cm^3^, excluding materials consisting of particles with a size lower than 1 nm. The VSSA of a material is generally calculated from its bulk density and its mass specific surface area. The latter is usually determined by gas absorption methodology called the BET-method [6] that allows surface area or porosity measurements as small as 1 nm. From a 3D reconstruction of a NM, its surface area and its volume can, in principle, be estimated directly, such that its VSSA can be calculated, even on a per particle basis. Advanced electron tomography methods were applied advantageously and successfully to characterize NM at a high resolution [4,5,7,8]. Most TEM-facilities do however not dispose of the required expensive equipment and lack the specialized expertise. Conventional electron tomography, where reconstructions are generated from a tilt series recorded in bright-field mode, using a single tilt axes with a tilt range up to ± 70°, becomes however a well-established technique. In this study, we examined the feasibility of three-dimensional visualization of silica and branched gold NM in suspension using conventional bright field (BF) ET. We examined whether such materials can be defined as a NM based on the measurement of their VSSA from its electron tomographic reconstruction. To evaluate the influence of missing wedge artifacts on the reconstruction and on the precision of the estimation of the surface area and volume of such NM, ET analyses of spherical colloidal gold nanoparticles were used as a control.

## Methods

Suspensions of spherical and branched gold NP were obtained from IMEC (Heverlee, Belgium). Aggregated silica nanomaterials NM-200 and NM-203 are supplied by the European Commission-JRC (Ispra, Italy) as representative reference NM. They are used as well at the OECD Working Party for Manufactured Nanomaterials programme as principal materials and international harmonization standards. The NM were brought on pioloform- and carbon-coated 400 mesh copper grids (Agar Scientific, Essex, England) that were pre-treated with 1% Alcian blue (Fluka, Buchs, Switzerland) to increase hydrophilicity, as described by Mast and Demeestere [9]. Gold NP were used undiluted. NM-200 and NM-203 were suspended in water containing 2% Fetal calf serum (PAA Laboratories GmbH, Pasching, Austria) at a concentration of 0.1 mg/ml and sonicated using a Vibracell™ 75041 sonicator (750 W, 20 kHz, Fisher Bioblock Scientific, Aalst, Belgium) with a 3 mm probe at 40% amplitude (10 W). A total energy of approximately 6200 J was added to the samples.

To obtain a maximal field of view, grids were mounted in a tomography holder (FEI, Eindhoven, The Netherlands) such that the squares were oriented diagonally with respect to the axis of the holder. Only objects in the centre of a grid square were analyzed using a Tecnai Spirit TEM (FEI) with a BioTWIN lens configuration and a LaB6-filament operating at an acceleration voltage of 120 kV.

Series of micrographs (tilt-series) were recorded semi-automatically assisted by the Xplore 3D tomography-module of the microscope control software (FEI) over a tilt range of at least 65°, or highest angle possible, at intervals of 1 degree. Shift and focus changes were corrected at every interval. Electron micrographs were acquired with a 4*4 K Eagle CCD-camera (FEI) at magnifications of 26,500 to 49,000 times and corresponding pixel sizes of 0.49 to 0.22 nm. The tilt series were aligned using the Inspect 3D software, version 2.5 (FEI) by iterative rounds of cross correlation until the alignment shifts were approaching to zero. Because of their higher signal to noise ratio, reconstructions using 10 to 20 iterations of the Simultaneous Iterative Reconstruction Technique (SIRT) algorithm were superior over reconstructions based on weighted back projection (WBP) and on the Algebraic Reconstruction Technique (ART) algorithm (not shown).

For visualization in 3D, the Amira software, version 4.1.2 (Mercury Computer Systems, France) was used. Isosurface rendering was used to compute a triangular approximation of the interfaces between the segmented sections. The segmentation was obtained based on a single threshold. This was chosen such that the obtained surface optimally matches the boundaries of the reconstructed orthogonal digital slices (orthoslices) of the NM in the xy-plane, where resolution is highest. The resulting surface was visualized using pseudo-coloring. To reduce missing wedge artifacts, so-called streaks, the surface was smoothed using a 2 × 2 × 2 averaging of voxels (downsampling). Using the 'Create Surface' function of Amira, a surface was derived from the isosurface, which allowed measurement of the surface area of the reconstructed 3D objects and of their enclosed volume.

Two-dimensional parameters of the reconstructed NP were measured from the TEM micrographs taken at 0° using the AnalySIS Solution of the iTEM software (Olympus, Münster, Germany). Briefly, contrast and brightness of the micrographs were optimized, the involved particles were enclosed in a frame (region of interest) and thresholds were set to separate particles from the background based on their electron density and size. The surface area and volume of individual spherical particles were approximated by the formulas to calculate the surface area (4πr^2^) and the volume (4/3πr^3^) of a perfect sphere, where r is replaced by the measured ECD of the projected particle divided by two. The sphericity, describing the 'roundness' of a particle by using central moments, was used to assess the hypothesis that the particle is a sphere in reality.

To measure the strength of correlation between the calculated VSSA and the measured VSSA, the nonparametric Spearman rank order correlation test was calculated using the SigmaPlot software, version 11.0 (Cosinus Computing B.V., Drunen, The Netherlands). To test the hypothesis that the mean VSSA obtained from ET reconstructions equals the threshold of 60 m^2^/cm^3^, the one-sample t-test (Sigmaplot) was used.

## Results

### ET of spherical gold nanoparticles

**Figure 1:**
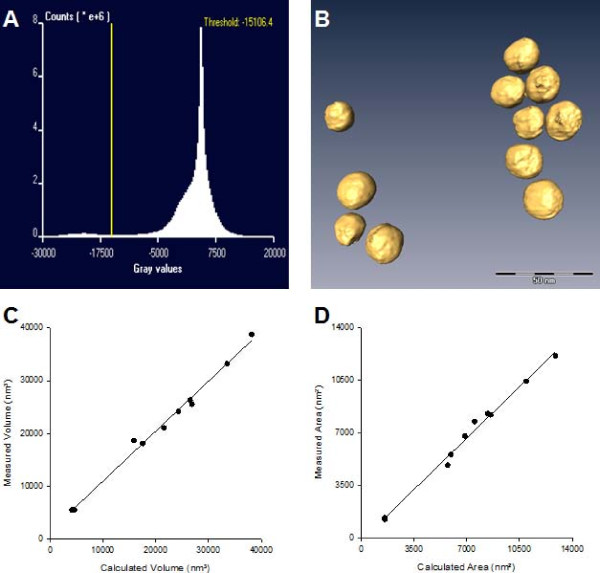
**Electron tomographic analysis of spherical gold nanoparticles**. Figure 1A represents the micrograph gray value range that served for setting the threshold. The threshold was set at -15106.4, that is somewhere between the two peaks. Figure 1B shows a representative electron tomographic 3D-reconstruction of spherical gold NP. Bar: 50 nm. Figure 1C and Figure 1D show the correlation between the calculated and measured volumes and areas of ten electron tomograms.

Electron tomographic reconstruction allowed visualizing the spherical gold NP in three dimensions (Figure 1B). The particles measure approximately 20 nm in diameter while the general morphology of all examined gold NP was almost spherical. Some small extensions of the surface were observed at the polar regions of the reconstructed particles. Local flattening was observed in the equatorial regions. The latter coincided with small zones in the original micrographs showing diffraction contrast, indicative for a confined crystalline organization. In the original micrographs taken at a tilt angle of 0°, the outline of the particles was roughly circular, although angular regions corresponding with a local crystalline structure were observed in certain particles.

From the isosurface based volume rendering of the ET reconstructions, the total surface area and volume of their composing gold particles could be measured. For example, the total surface area and the volume of the NP shown in Figure 1B are 13,895 nm^2 ^and 38,763 nm^3^, respectively. This corresponds with a VSSA of 332 m^2^/cm^3^. The mean VSSA ± SEM, determined from 10 ET reconstructions (Table 1), is 316 ± 7 m^2^/cm^3^, which is significantly different (P < 0.05) from 60 m^2^/cm^3^.

**Table 1:** Mean volume specific surface area of different nanomaterials based on electron tomographic reconstructions

Type of nanomaterial	n	Volume-specific surface area (m^2^/cm^3^)^a^
Spherical Gold	10	316 ± 7
Branched Gold	5	177 ± 29
Precipitated Silica (NM-200)	5	342 ± 36
Pyrogenic Silica (NM-203)	5	219 ± 23

^a ^Values represent mean VSSA ± SEM

The reconstructed gold particles showed no obvious elongation along their z-axis and image analysis of the transmission electron micrographs of the individual particles taken at a tilt angle of 0° resulted in a mean sphericity of 0.86. Hence, it was concluded that these gold particles are almost spherical and that their surface area and volume can be closely approximated by the formulas to calculate the surface area and the volume of a perfect sphere. Figure 1C and 1D show the correlations between the calculated and measured volume and surface area, respectively, for ten ET reconstructions consisting of one to 11 gold NP. Both for the volume and the surface area, the Spearman correlation coefficient was 0.98.

### ET of branched gold nanoparticles

**Figure 2:**
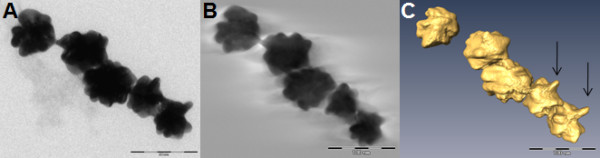
**Electron tomography of branched gold NP**. Figure 2A represents the original micrograph of five branched gold NP taken at 0°. Figure 2B is a 0.38 nm section through the reconstructed volume shown in Figure 2C. Figure 2C shows a representative electron tomographic 3D reconstruction of branched gold NP. Arrows indicate surface extensions. Bars: 100 nm.

Branched gold NP measure approximately 50 nm in diameter and show a highly irregular rather than a spherical morphology: they are characterized by their surface extensions or peaks. These features can be deduced from 2D images, like the original micrograph (Figure 2A) and the orthoslices through the reconstruction (Figure 2B). Under certain orientations, and for a few images of the tilt series, diffraction contrast contributed considerably to the image formation of the extensions of branched gold particles, suggesting zones with a crystalline organization. Nevertheless, the resolution of the final ET reconstruction remained high enough to visualize the branched gold NP in three dimensions (Figure 2, Additional file 1), where their surface topology can be interpreted easier than in the 2D images. The surface area and volume of the branched gold nanoparticles were measured for five ET reconstructions such that VSSA could be calculated (Table 1). The mean VSSA ± SEM is significantly different (P < 0.05) from 60 m^2^/cm^3^.

### ET analyses of silica NM

**Figure 3:**
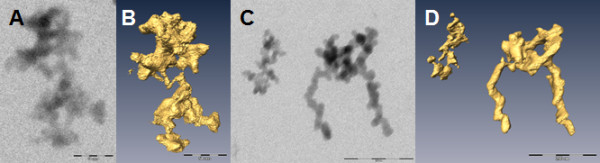
**Electron tomographic analyses of silica NM**. The micrographs, taken at 0°, show one (Figure 3A) and two aggregates (Figure 3C) consisting of multiple primary subunits of NM-200 and NM-203, respectively. Figure 3B and Figure 3D show the corresponding ET reconstructions. Bars: 200 nm.

It is not evident to envisage the structure of the silica reference materials NM-200 and NM-203 appropriately by conventional bright field TEM (Figure 3A and 3C). Their relatively low molar mass results in a low contrast, while their complex morphology results in blurring of ultrastructural details due to superposition of projected features. Electron tomographic reconstruction in three dimensions circumvents these difficulties. Figure 3B and 3D, and the corresponding Additional files 2 and 3, illustrate that both the precipitated silica NM-200 and the pyrogenic silica NM-203 consist of aggregates of very complex morphology composed of a variable number of interconnected primary subunits. Although the site where an aggregate interacts with the grid can be found in the 3D reconstruction as a relatively flat surface, structures of primary subunits remain extended in the z-direction, resulting in similar dimensions along the three axes. This suggests a limited flexibility of the material. Measurement in 3D space showed that individual aggregates in both NM-200 and NM-203 are composed of similarly sized primary subunits. The size of the subunits of the aggregates of NM-200 is relatively constant: they measure approximately 20 nm in diameter. The size of the subunits of different aggregates of NM-203 is variable: the subunits of the left aggregate shown in Figure 3D measure, for example, 8 to 12 nm in diameter, while the subunits of the right aggregate measure approximately 20 nm in diameter. In any of the tilt series of NM-200 and NM-203, diffraction contrast was observed, confirming their amorphous structure. The surface area and volume of NM-200 and NM-203 were measured for five ET reconstructions and the VSSA was calculated (Table 1). For both materials, the mean VSSA were significantly different (P < 0.05) from 60 m^2^/cm^3^.

## Discussion

By electron tomographic reconstruction based on conventional BF TEM, the general morphology of gold and silica NM was visualized in 3D. In orthoslices of the examined NM in the xy-plane, as presented in Figure 2B, the surface can readily be distinguished from the background and from missing wedge artifacts, like streaks. In such orthoslices, the surface features of a NM can be seen and measured without interference of higher or lower lying structures inherent to conventional TEM.

Segmentation by isosurface rendering allows accessing the 3D information of an ET reconstruction in greater detail than digital slicing. Such 3D visualization and measurement of the surface features of NM can contribute to bring the second condition of the definition of a nanomaterial proposed by the European Commission [2] in practice: structures in one or more dimensions in the size range of 1-100 nm can be shown.

From the 3D reconstructions, the surface area and the volume of the examined NM could be estimated directly and the VSSA was calculated. The mean VSSA of all examined NM was significantly larger than the threshold of 60 m^2^/cm^3 ^such that these materials can be classified as NP according to the third condition of this definition. As opposed to the BET-method [10], ET is not limited to powders and/or dry solid materials: it can be applied to a large variety of NM samples, including suspensions of complex particles, provided that the material can be suitably coated on an EM-grid.

To optimally characterize the morphology of a NM by ET reconstruction, it is required that (i) the projection requirement is met [4]; (ii) missing wedge artifacts are minimal and (iii) isosurface rendering optimally fits the NM surface.

Our results indicate that, in principle, the characterization and definition of NM can benefit from application of conventional BF ET. In the scope of putting this technique in practice for the characterization and definition of gold and silica NM, following approach is suggested to reconcile the limitations of conventional BF ET with the above-described conditions.

(i) The projection requirement states that for an image intensity to be usable for ET reconstruction, it has to be a monotonic function of a projected physical quantity [4]. The examined silica NM were shown to be amorphous and weak scattering such that their mass thickness is the dominant contrast mechanism. The BF images of the tilt series are thus essentially projections on which tomographic reconstructions can be based [5]. In the branched gold particles, and to a small extent in the spherical gold particles, diffraction contributed to image formation and the projection requirement is not fulfilled for the entire tilt series. Certainly, a combination of scanning transmission electron microscopy (STEM) and high angle annular dark field imaging (HAADF) which is insensitive to Bragg diffraction will be preferable over bright field imaging to visualize these NM at high resolution [11-13]. Because diffraction increases the background of the reconstruction and reduces its resolution, BF ET has been suggested to be of only limited value to analyze crystalline nanostructures [11,12]. However, Ahrenkiel et al. [14] argumented that conventional BF ET still can provide useful information on the structure of particles with relatively small crystallite size if suitable acquisition conditions are chosen. Hence, the examined material was not embedded to assure positive contrast originating from the specimen at all orientations, while diffraction contrast was minimized while preserving some mass-thickness contrast by using a large objective aperture.

(ii) An important physical limitation of electron tomography arises from the fact that finite specimen thickness and tilt geometry within an electron microscope column prevent the collection of projection images spanning a complete angular range (± 90° tilt series). This results in a "missing wedge" of information in reciprocal space and results in anisotropic resolution in the resulting reconstruction [7]. Only for specific samples that were properly shaped using a focused ion beam and mounted in a special holder, these subsampling effects could be avoided [15]. The missing wedge can be reduced by collecting a so-called dual-axis tilt series in two mutually orthogonal directions [7,16-18] such that a missing pyramid is obtained. Because above-described approaches require high technicity and are unpractical for routine analyses, the missing wedge artifacts of the reconstructions of the silica and gold NM were only reduced using a small tilt increment and maximizing the tilt range of the electron tomogram. Because gold and silica NM are hardly sensitive to radiation damage, extensive data collection using a high frequency of imaging can be applied. Because our software and hardware limit the amount of data that can be aligned and reconstructed in a timely manner, 4*4 K micrographs were taken with one degree intervals and datasets were reduced to contain only few particles. New developments of soft- and hardware and GPU-based ET implementation [19] promise faster data processing allowing a combination of smaller intervals and the simultaneous analysis of several hundreds of particles under similar imaging conditions. Moreover, improvement of the quality of reconstructions seems possible by using newly developed reconstruction algorithms like the discrete algebraic reconstruction technique (DART) which suffers less from missing wedge artifacts than SIRT [8]. In our hands, the correction of the tilt axis during alignment appeared very important to reduce reconstruction artifacts like streaks. When the axes were not corrected accurately, the streaks were included in the particle volume resulting in an elongation of the particles which was at least as strong as the missing wedge dependent elongation described in [4].

(iii) In the examined NM, the value of the threshold was selected such that the obtained surface optimally matches the boundaries of the reconstructed orthogonal digital slices (orthoslices) of the NM in the xy-plane, where resolution is highest. This threshold value was in general close to the minimal value between both peaks of the bimodal curve (Figure 1A) of the histogram representing the number of voxels in function of their grey value. In the future, computational techniques that determine the optimal grey value for thresholding can allow more efficient segmentation, eliminating the subjectivity associated with manual segmentation [20].

To evaluate the influence of missing wedge artifacts on the reconstruction and, in particular, on the validity of the quantitative results obtained from the reconstructions, the ET based analyses of colloidal gold nanoparticles with a known spherical morphology were evaluated as a control. The high correlation (r = 0.98) between the measured values of area and volume and the areas and volumes calculated from the ECD of projected NP indicates that the values measured from electron tomographic reconstructions are valid for these gold particles. On this basis, it is assumed that the surface and volume measures of the branched gold and silica NM, which lack symmetry, can be relied on also. However, it has to be stressed that the absolute numbers presented in Table 1 should be interpreted with caution since they are based on a very low number of observations. This table illustrates the possibilities of the method in principle but it remains unsure whether the selected particles are representative for the entire examined samples. The latter requires, at least, the ET analysis of larger numbers of randomly selected particles. The evaluation of the VSSA values measured using methods like ET, BET or small-angle X-ray scattering (SAXS), and their corresponding uncertainties and limitations requires a dedicated study. For NM-200, the VSSA estimated by ET (342 ± 36 m^2^/cm^3^) is higher than the value obtained by SAXS (270 ± 17 m^2^/cm^3^, personal communication Camille Guiot, French Atomic Energy and Alternative Energies Commission, France), but lower than the values obtained by BET (435 m^2^/cm^3^, personal communication Rosica Petrova, Institute of Mineralogy and Crystallography - Bulgarian Academy of Sciences, Bulgaria). For NM-203, the VSSA estimated by ET (219 ± 23 m^2^/cm^3^) is lower than both the VSSA obtained by SAXS (367 ± 30 m^2^/cm^3^, personal communication Camille Guiot) and BET (469 m^2^/cm^3^, personal communication Rosica Petrova).

The EU definition expects a resolution of at least 1 nm [2]. It is doubtful whether this resolution can be obtained routinely by BF TEM [21]. For an exact description of the physical characteristics of a NM, atomic resolution within a reconstruction can be important. It is however not an absolute prerequisite to apply the definition if it is taken into account that lack of resolution results in an underestimation of the VSSA because, relatively, nanometer-sized surface features contribute more to an increase of the particle surface than to an increase of its volume.

## Conclusion

As a proof of principle, it was shown that application of conventional BF ET allows 3D visualization of the examined gold and silica NM and allows measuring their surface features and VSSA. This approach can hence contribute to bring the second and third condition of the definition of a nanomaterial proposed by the European Commission in practice [2]. Recent technical developments promise for the near future the possibility to analyze large numbers of particles [19] representative for the sample, a better reconstruction [8] and less influence of missing wedge artifacts [7,16-18] such that the characterization of nanomaterials by transmission electron tomography can become more precise and less time-consuming.

## Competing interests

The authors declare that they have no competing interests.

## Authors' contributions

EVD and JM contributed equally to this manuscript. EVD produced most of the electron tomograms and the illustrations. JM developed the basic concept and took care of the redaction of the manuscript. PJD assisted in sample preparation and MAF assisted in the alignment and reconstruction and visualization of electron tomograms. All authors have read and approved the final manuscript.

## Supplementary Material

Additional file 1 **Electron tomographic reconstruction of branched gold NP**. The video shows a surface rendered view of the branched gold NP shown in Figure 2C. Observe the surface extensions. Bar: 50 nm. Click here for file

Additional file 2 **Electron tomographic reconstruction of nanostructured silica nanomaterial NM-200**. The video shows a surface rendered view of the nanostructured silica NP shown in Figure 3B. Bar: 40 nm. Click here for file

Additional file 3 **Electron tomographic reconstruction of nanostructured silica nanomaterial NM-203**. The video shows a surface rendered view of the nanostructured silica NP shown in Figure 3D. Bar: 50 nm. Click here for file
